# Magnetic Nanoparticle-Based Electrochemical Sensing Platform Using Ferrocene-Labelled Peptide Nucleic Acid for the Early Diagnosis of Colorectal Cancer

**DOI:** 10.3390/bios12090736

**Published:** 2022-09-07

**Authors:** Simge Balaban Hanoglu, Ezgi Man, Duygu Harmanci, Serife Tozan Ruzgar, Serdar Sanli, Nazim Arda Keles, Atakan Ayden, Bilge Guvenc Tuna, Ozgul Duzgun, Omer Faruk Ozkan, Soner Dogan, Faezeh Ghorbanizamani, Hichem Moulahoum, Emine Guler Celik, Serap Evran, Suna Timur

**Affiliations:** 1 Department of Biochemistry, Faculty of Science, Ege University, Bornova, Izmir 35100, Turkey; 2 Central Research Test and Analysis Laboratory, Application and Research Center, Ege University, Bornova, Izmir 35100, Turkey; 3 Department of Medical Biology, School of Medicine, Yeditepe University, Istanbul 34755, Turkey; 4 Department of Biophysics, School of Medicine, Yeditepe University, Istanbul 34755, Turkey; 5 Department of Surgical Oncology, University of Health Sciences, Umraniye Training and Research Hospital, Istanbul 34764, Turkey; 6 Department of Bioengineering, Faculty of Engineering, Ege University, Bornova, Izmir 35100, Turkey

**Keywords:** biosensor, point-of-care diagnostic (POC), magnetic nanoparticles, colorectal cancer (CRC), blood biomarkers

## Abstract

Diagnostic biomarkers based on epigenetic changes such as DNA methylation are promising tools for early cancer diagnosis. However, there are significant difficulties in directly and specifically detecting methylated DNA regions. Here, we report an electrochemical sensing system based on magnetic nanoparticles that enable a quantitative and selective analysis of the methylated septin9 (mSEPT9) gene, which is considered a diagnostic marker in early stage colorectal cancer (CRC). Methylation levels of SEPT9 in CRC samples were successfully followed by the selective recognition ability of a related peptide nucleic acid (PNA) after hybridization with DNA fragments in human patients’ serums and plasma (*n* = 10). Moreover, this system was also adapted into a point-of-care (POC) device for a one-step detection platform. The detection of mSEPT9 demonstrated a limit of detection (LOD) value of 0.37% and interference-free measurement in the presence of branched-chain amino acid transaminase 1 (BCAT1) and SRY box transcription factor 21 antisense divergent transcript 1 (SOX21-AS1). The currently proposed functional platform has substantial prospects in translational applications of early CRC detection.

## 1. Introduction

Nanomaterial-based electrochemical biosensors are widely used because they increase signal strength, facilitate the immobilisation of biorecognition materials, and simplify sample preparation [1]. It is also possible to use these systems by adapting them to point-of-care systems (POC) at bed-side applications [2]. While these nanomaterials protect the active areas of sensor systems, they also facilitate surface modification with recognition elements such as antibodies and ssDNA [3]. In addition, they play a key role in optimisation studies by providing a balance between capture and catalytic capabilities [4]. Magnetic nanoparticles (MNPs) are among the nanostructured materials that have superparamagnetic properties and provide various functional groups that can be modified with different biomolecules [5]. In particular, iron-based nanoparticles, which were also used in this study, can be easily immobilised on the surface using magnets in biosensing studies due to their strong magnetic properties compared to other nanoparticles [6]. Moreover, the interaction of the target biomolecule with these MNPs ensures that it is separated from the impurities, eliminating the matrix effect. They have a high surface area and additional anisotropy contributions [7], as well as providing a disposable detection surface and increasing the detection sensitivity and stability of sensor systems [8,9].

According to the World Health Organization (WHO), colon cancer or colorectal carcinoma (CRC) is the third highest mortality-causing cancer worldwide [10,11]. The mechanism of CRC starts from a noncancerous polyp that develops inside the colon and rectum, eventually leading to cancer [12]. Rapid diagnosis of this disease affects the chances of survival, quality of life, and cost of medical treatment [13,14,15]. Various methods such as faecal occult blood test (FOBT), colonoscopy, periodic computed tomography (CT), biopsy, and serum carcinoembryonic antigen (CEA) measurement are presently used in CRC screening [6]. Some of these methods require invasive procedures, technical equipment and specialists. Although FOBT is a non-invasive and inexpensive method, its sensitivity is limited [16]. Even though colonoscopy is the gold standard for CRC screening, it requires time-consuming operation steps and sometimes causes serious complications [17]. Concerning CT, it has limited sensitivity, especially for small lesions, and could cause a high rate of false positivity [18]. As for the CEA testing as another biomarker, the sensitivity and specificity are not optimal [19]. Hence, there is a need to discover new markers for the development of novel methods of diagnosis, prognosis, and treatment. A biomarker is a molecular pattern that can be used in both diagnosis and treatment. The biomolecules are classified as diagnostic, prognostic, or predictive and play an important role, especially in the early diagnosis of CRC [20]. The well-known genetic biomarkers associated with CRC are V-Ki-ras2 Kirsten rat sarcoma viral oncogene (KRAS), B-type Raf kinase (BRAF), adenomatous polyposis coli (APC), phosphoinositide 3-kinases (PI3K), phosphatase and tensin homologue (PTEN), and TP53 protein [21]. In addition, epigenetic biomarkers have also been discussed for many years [22,23]. One of the most popular and studied issues is DNA methylation. Hypermethylation in a promoter region or tumour suppressor gene could lead to the inactivation of that gene. The consequences of this differentiation are changes in cellular activity that pave the way for cancer development. Research has shown that DNA methylation, particularly hypermethylation of the septin 9 (SEPT9) gene, is one of the most common abnormal epigenetic changes that plays a fundamental role in the development and progression of CRC [24]. Basically, any SEPT9 gene methylation assay aims to detect the abnormal methylation in the promoter region of SEPT9 gene DNA released from CRC cells into the peripheral blood [25]. Hypermethylation of the CpG island in the promoter region has been found to lead to loss of tumour suppressor function and promote the development of CRC [26]. When the promoter region of the SEPT9 gene is hypermethylated, the DNA of the gene is released from necrotic and apoptotic cancer cells into the peripheral circulating blood during the carcinogenesis of CRC. Therefore, the risk of CRC can be determined by detecting the degree of DNA methylation of the specific promoter region of the SEPT9 gene in peripheral blood [25]. Studies have shown that the most important feature that distinguishes SEPT9 from currently used glycoproteins such as CEA, CA199, CA242, CA72-4, and CA125 is that it is the most sensitive diagnostic biomarker that can be used alone for the early diagnosis of CRC [20,25].

Recently, an electrochemical biosensor with peptide nucleic acids (PNAs) as bio-recognition elements has been developed [27]. PNAs are DNA analogs in which the negatively charged sugar-phosphate backbone has been completely replaced by a neutral “peptide-like” backbone [28]. Due to its uncharged backbone, PNA could hybridise with the target DNA and RNA chains with high selectivity [27]. On the other hand, it is possible to modify the ends of the “PNA capture probes” that selectively bind to the target DNA or RNA fragment with the redox-active metal complex like ferrocene (Fc). Hence, the use of an electroactive label enables the design of an electrochemical sensing platform [29,30]. Taken together, the current study proposes the development of an electrochemical sensing surface based on a gene-based biomarker detection biosensor for the early diagnosis of CRC. The proposed platform was based on the use on MNPs, 5-methylcytosine (5-mC) antibody, and a hybridization system (Fc-PNA) to detect the methylation levels of the specific CRC biomarker mSEPT9. After optimization and characterization of patients’ serum and plasma samples, the sensing system was adapted onto a POC device. The proposed biosensors’ clinical and translational applications for mSEPT9 analysis in CRC patients demonstrated a great potential for future development.

## 2. Materials and Methods

### 2.1. Chemicals and Reagents

All chemicals and reagents used in this study are of analytical grade. A 5-methylcytosine (5-mC) antibody was purchased from Abcam PLC (Cambridge, UK). Tetraethyl orthosilicate (TEOS), 3-aminopropiltrietoxysilane (APTES), N-(3-dimethylaminopropyl)-N′-ethylcarbodiimide (EDC), 2-(N-morpholino)ethanesulfonic acid (MES), N-hydroxysuccinimide ester (NHS), bovine serum albumin (BSA) (lyophilised, ≥96%), potassium chloride, potassium hexacyanoferrate (III) (HCF), and human serum (H4522) were procured from Sigma-Aldrich (Schnelldorf, Germany). The MethylFlash™ Methylated DNA Quantification Kit (Fluorometric) was purchased from EpiGentek Group (Farmingdale, NY, USA). The OneStep qMethyl-PCR Kit (D5311 and D5310) was purchased from Zymo Research (Tustin, CA, USA). Cobas^®^ cfDNA Sample Preparation Kit was provided by La Roche Ltd (Basel, Switzerland).

The amplicon 5 sequence of SEPT9 was obtained from the UCSC Genome Browser (https://genome.ucsc.edu/ 4 January 2021). The promoter sequences of the branched-chain amino acid transaminase 1 (BCAT-1) and SRY box transcription factor 21 antisense divergent transcript 1 (SOX21-AS1) genes were retrieved from the Eukaryotic Promoter Database (https://epd.epfl.ch/ 4 January 2021). Oligonucleotides were purchased from Eurofins (Ebersberg, Germany) and Ella Biotech (Fürstenfeldbruck, Germany). The ferrocene-labelled peptide nucleic acid (Fc-PNA) was purchased from PNABio (Thousand Oaks, CA, USA) with the following sequence Fc-O-CCCCTCAAGTTCCCA-NH_2_, where the ferrocene moiety and ethylene glycol linker are denoted by Fc and O, respectively.

### 2.2. Instrumentation

The PalmSens 4 potentiostat (Palm Instruments, Houten, The Netherlands) was used for electrochemical measurements such as differential pulse voltammetry (DPV), cycle voltammetry (CV), and impedance spectroscopy (EIS). CV and EIS measurements were performed for surface characterisation, while DPV measurements were used to determine analytical characteristics. Screen-printed carbon electrodes (SPCEs) (DropSens-DRP-150) were provided by Metrohm (Herisau, Switzerland). Neodymium magnets (4.0 mm diameter) were purchased from Megahand Information Technologies (Izmir, Turkey). Scanning electron microscopy (SEM, Thermo Fischer Scientific Apreo S LoVac model, Hillsboro, Oregon, USA) and dynamic light scattering (DLS) (Malvern Zetasizer, NanoZS model, Malvern Instruments Ltd., Worcestershire, Malvern, UK) were used to characterise the MNP-antibody conjugates. In addition, the magnetic moment of MNPs was determined with a vibrating-sample magnetometer (VSM) (Lake Shore, Model 7407). For the dsDNA measurements, Thermo Fisher Scientific NanoDrop ONE device was used. Electrochemical measurements were performed in an electrochemical cell containing 0.1 M KCl and 5.0 mM HCF in 1X PBS buffer (pH 7.4) for measurements of SPCE/MNP; SPCE/MNP/EDC/NHS; SPCE/MNP/EDC/NHS/5-mC Ab steps. The electrochemical cell containing 0.1 M KCl in 1X PBS buffer (pH 7.4) was used for measurement after Fc-PNA addition.

### 2.3. Collection of the Clinical Samples

Blood samples were collected from a total of 10 CRC patients who were admitted to the Umraniye Training and Research Hospital, Istanbul, Turkey and 10 healthy individuals as controls. The samples were then transferred and processed at the Department of Medical Biology, Yeditepe University, School of Medicine, Istanbul, Turkey. The CRC patients were histologically confirmed. Blood samples from CRC patients were obtained prior to therapy (pre-therapeutic samples). The study protocol was approved by the Umraniye Training and Research Hospital clinical research ethics committee (no: 2020/351). All patients had provided written informed consent to be included in the study.

### 2.4. Isothermal Titration Calorimetry

Isothermal titration calorimetry (ITC) experiments were performed with PEAQ-ITC (Malvern Panalytical, UK) at 25 °C. The reference power was set to 10 µcal/s. After degassing, 300 µL of the DNA solution (5.0 µM) was loaded into the reaction cell and 40 µL of the PNA solution (5.0 µM) was loaded into the syringe. The stirring speed was set to 750 rpm. The titration was performed with 150 s spacing time. The data were analysed with PEAQ-ITC software. The dissociation constant (K_d_), enthalpy change (ΔH) and Gibbs free energy change (ΔG) were determined.

### 2.5. MNP Synthesis, Bioconjugation, and Hybridization

MNPs were synthesised according to the procedure described by Sanli et al. [6]. Briefly, FeCl_3_ (2.0 M) and FeCl_2_ (1.0 M) solutions were mixed and then NH_4_OH solution (25%, 10 mL) was added dropwise, and the solution was incubated for 30 min. The obtained MNPs were dried at 80 °C for 6 h after washing with distilled water and ethanol.

For amino modification of MNP, 300 mg of MNPs were added to a solution consisting of 80% ethanol (100 mL) and ammonium solution (2.5 mL). TEOS solution (5.0 mL) was added after sonication. The solution was incubated at 40 °C for 4 h and washed with methanol. Then, APTES (5.0 mL) and methanol (100 mL) were added to the MNPs and incubated at 60 °C for 6 h, after sonication for 15 min. The obtained MNPs were washed and dried at room temperature. The characterization of MNPs was performed by scanning electron microscopy. The dynamic size of the synthesised MNPs was analysed by dynamic light scattering (DLS) [31].

MNP conjugation with 5-mC Ab was performed according to the method of Singh et al. [32]. Then, 10 mg EDC, 1.7 mg NHS, and 0.5 mg MNPs were suspended in MES buffer (0.1 M, pH 6.0) and the solution was incubated at 1000 rpm, 10 min at RT. The MNPs activated with amino groups were collected with a neodymium magnet. After the washing steps, the MNPs were resuspended with 250 µL of PBS buffer (pH 7.4). Then, 50 µL of MNPs and 10 µg/mL of 5-mC Ab were mixed to a final concentration of 300 µL. The solution was incubated at 1000 rpm for 2 h at ambient conditions. The MNP/5-mC Ab conjugate was collected with a magnet and washed twice with 1.0% BSA solution before being resuspended again in 1.0% BSA solution. The conjugate was freshly prepared before use.

The Hybridization of Fc-PNA with mSEPT9 Fragment was performed by first subjecting mSEPT9 fragments to heat treatment to avoid any possible self-hybrids and ensure effective hybridization with Fc-PNA. For this, 20 nM mSEPT9 fragments (0; 25; 50; 75 and 100% methylation levels) were prepared in 1X PBS buffer. The fragments were then incubated at 95 °C for 5.0 min and left at RT for 15–20 min to cool. Hybridization buffer (10 mM Na_2_HPO_4_, 10 mM KH_2_PO_4_, 1.0 M NaCl and 20 mM MgCl_2_ in 1X PBS buffer) was added to the folded mSEPT9 fragments to a final concentration of 10 nM [33]. A final concentration of 0.5 µM Fc-PNA was incubated for 1 h at RT for hybridization [3].

### 2.6. Sensor Design

SPCEs were used to design the biosensor platform and a neodymium magnet was attached to the back of the working electrode of the SPCEs. Then, 10 µL of MNP/5-mC Ab conjugate was added to the surface of the working electrode, and after drying, 5.0 µL of mSEPT9/Fc-PNA solutions were applied to the electrode surface. The electrode was incubated at RT for 1.0 h. Electrochemical measurements (CV, DPV, EIS) were performed after each modification step. CV measurements with a potential range between -0.4 V and 0.8 V was determined at a scan rate of 50 mV/s, while the potential range for DPV measurements was set at −0.4 V to −0.8 V. EIS measurements were performed from 0.02 Hz to 10 Hz at 0.18 V. Electrochemical measurements indicating the surface modification and binding after each steps (Bare SPCE; SPCE/MNP; SPCE/MNP/EDC/NHS; SPCE/MNP/EDC/NHS/5-mC Ab) were performed in a cell containing 0.1 M KCl and 5.0 mM HCF in 1X PBS buffer (pH 7.4), whereas the measurement after the addition of the mSEPT9/Fc-PNA hybridised solution was performed in a cell containing 0.1 M KCl in 1X PBS buffer (pH 7.4). In this case, the presence of Fc caused an electrochemical signal response, unlike the other steps in which HCF was followed as a redox probe.

### 2.7. Optimization and Calibration Graph

Various amounts of 5-mC Ab (1.0, 5.0, 10, and 15 µg/mL) were tested for the optimization of bioconjugate preparation with MNPs. The resulting bioconjugates were then dropped and stabilised onto the working electrode. Then, DPV measurements were performed after the addition of mSEPT9/Fc-PNA (50%). Subsequently, the concentration of mSEPT9 fragments was optimised. For this purpose, different concentrations of 100% mSEPT9 fragment (1.0, 5.0, 10, and 50 nM) were hybridised with the Fc-PNA probe. In addition, 30-, 45-, 60-, and 75-min incubation times were tested for the optimization after the addition of mSEPT9-Fc-PNA.

A calibration curve was drawn using different methylation percentages of SEPT9 fragments (0; 25; 50; 75; and 100%). mSEPT9 fragments were hybridised with Fc-PNA and DPV measurements were performed. The current difference between the MNP/5-mC Ab with mSEPT9/Fc-PNA (∆µA) was calculated and the current differences vs. mSEPT9 were subtracted from the current difference obtained for 0% mSEPT9. A calibration curve was generated between current signals and analyte amount.

### 2.8. Interferences

Methylated BCAT1 and SOX21-AS1 fragments were selected as interferences that could freely circulate together with SEPT9 fragments. BCAT1 and SOX21-AS1 fragments were initially mixed and applied to the sensor platform to detect the selectivity towards SEPT9.

### 2.9. Sample Application and Confirmation

Commercial human serum was used to determine the effects of the sample matrix. mSEPT9 fragments (50% and 75% methylation) were added to the human serum and sensor responses were registered after hybridization of Fc-PNA.

For the sample application, initially cfDNAs were isolated from plasma samples (numbers of CRC patient samples and healthy samples are both 10) according to the manufacturer’s instructions (cfDNA Sample Preparation Kit, Roche). The concentration and purity of cfDNA were determined using Nanodrop. Subsequently, the concentration of cfDNAs was adjusted and hybridized with Fc-PNA as described in Section 2.6. cfDNA/Fc-PNA solutions were applied to the electrode surface functionalised with MNP/5-mC Ab.

All data were confirmed using the Methylated DNA Quantification Kit (ELISA) and the OneStep qMethyl-PCR Kit.

### 2.10. POC Design

In the final part, a circuit board was designed to fabricate a practical POC device for electrochemical diagnosis of CRC by detecting mSEPT9 as a biomarker. The circuit elements used are shown in Figures S1 and S2. The electrode data was calculated on the computer using an application written in Python. The raw graphical data obtained with the developed POC device were sent to the mobile device using the BlueDevil open-source communication protocol and the Linux-based application via the Bluetooth module.

### 2.11. Data Analysis

Data were analysed using GraphPad Prism Statistical Package version 5.0 software. Differences in methylation change between the two groups were tested using the Mann–Whitney test. The Spearman correlation coefficient method was used to evaluate the association between the result of PCR, ELISA and the developed sensor. Quantitative data were expressed as data plots with mean ± SD. A *p*-value < 0.005 was considered significant.

## 3. Results and Discussion

Septin proteins are biomolecules involved in many cellular processes. Epigenetic changes associated with SEPT9 are currently being investigated primarily in cancer research. Several studies have also shown that hypermethylation is observed in colorectal cancer [26]. While alterations in the SEPT9 gene alone have a diagnostic impact on the clinical approach, the determination of SEPT9 hypermethylation has both a diagnostic and prognostic impact on the clinical process. The EpiproColon kit is routinely used for the detection of SEPT9 in blood [29]. However, the fact that these systems require preliminary studies and have long phases increases the need for novel alternatives. Other drawbacks include the multi-step and time-consuming processes, the need for experts, the high cost, and the incompatibility with miniaturization. It is very important to effectively detect and measure biomarkers at different stages of the harvest by overcoming the limitations of routine methods, especially in diseases where early diagnosis is very important, such as cancer. There is a need for the design and fabrication of POC systems that provide reliable measurements at the bedside. From this point of view, electrochemical biosensors are a promising alternative to conventional methods due to their unique properties, portability, and miniaturization, as they respond in real-time and are able to detect tumour biomarkers of different types, specifically and simultaneously.

**Scheme 1:**
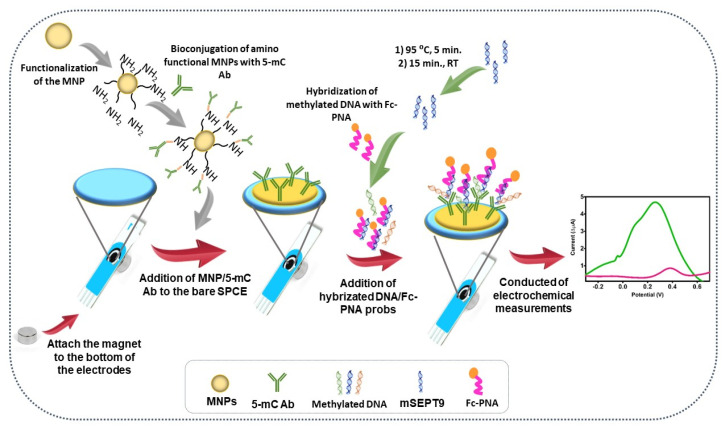
Step by step surface design and the analysis principles.

Here, we developed an electroactive surface to detect the methylation content of the SEPT9 fragment in CRC samples. Initially, MNPs were synthesised, and functionalised with amino groups. Afterwards, the carboxyl group of 5-mC Ab was conjugated to amino-functional MNP via EDC/NHS chemistry and the resulting conjugate was dropped and stabilised onto the SPCE surface by the means of magnetic force. In addition, SEPT9 fragments with different levels of methylation were hybridised with Fc-PNA. The mixture was then deposited over the working electrode. The preparation steps of the biosensing surface are shown in Scheme 1.

### 3.1. ITC Results

Amplicon 5 of SEPT9 was targeted in this study, as it was shown to be related to the cancer-associated increase in methylation [34]. A 60-nucleotide region with 8 possible cytosine methylation sites was chosen as the target sequence. The target sequence with 8 methylated cytosines was named 100met_SEPT9 to identify the 100% methylation. Accordingly, other sequences with 6.0, 4.0, and 2.0 methylated cytosines were named 75met_SEPT9, 50met_SEPT9, and 25met_SEPT9, respectively. Since hypermethylated BCAT1 and SOX21-AS were shown to be significant in cancer [35,36], they were chosen as interferents. The sequences are shown in Table S1.

#### Thermodynamic Features of the PNA-DNA Interaction

ITC was used to investigate the interaction between PNA and the methylated SEPT9 DNA fragments. The obtained ITC curves are shown in Figures S3–S7. Although some minor differences were observed, PNA displayed K_d_ values in the nM range against both unmethylated and methylated SEPT9 fragments (Table S2). As suggested by Alan et al. [37], this finding confirms the strong binding between PNA and the target DNA sequences. PNA showed a slightly lower affinity for 100met_SEPT9. Nevertheless, PNA showed similar hybridization to the unmethylated and methylated SEPT9 fragments, which could be attributed to the selected site for PNA binding. PNA probe was designed according to the TGGGAACTTGAGGG with no methylated cytosines, which could have minimised the effect of methylation on hybridization.

### 3.2. Characterisation

**Figure 1:**
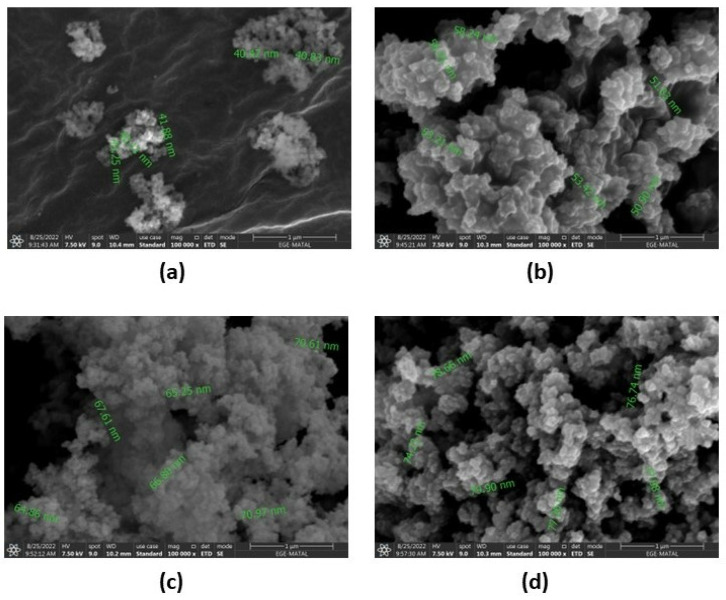
SEM images for (**a**) MNP, (**b**) MNP/TEOS, (**c**) MNP/TEOS/APTES, (**d**) MNP/TEOS/APTES/EDC/NHS/5-mC Ab.

**Figure 2:**
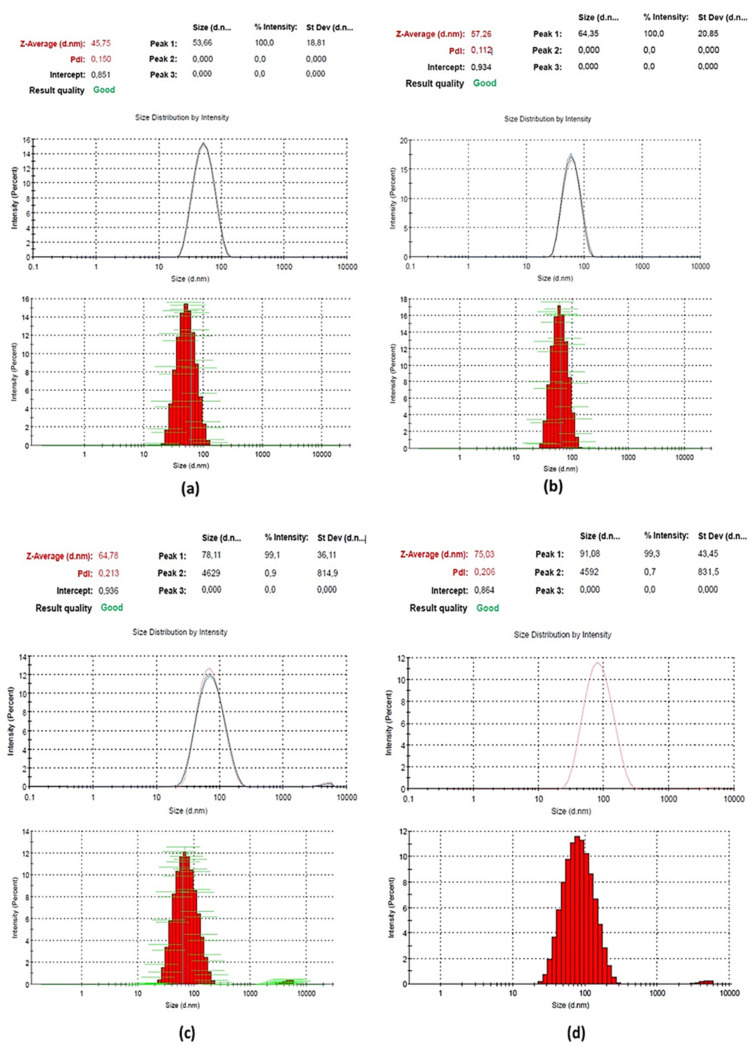
DLS histograms of (**a**) MNP, (**b**) MNP/TEOS, (**c**) MNP/TEOS/APTES, (**d**) MNP/TEOS/APTES/EDC/NHS/5-mC Ab.

Characterisation of the synthesised MNPs were performed using VSM measurements (Figure S8). The MNP (Ms = 52.3 emu/g) after modification with TEOS (MNP/TEOS) (Ms = 55.1 emu/g) samples showed similar magnetization properties. It was observed that the magnetism decreased after APTES was added to the MNP/TEOS structure (MNP/TEOS/APTES) (Ms = 43 emu/g). SEM and DLS were used after all modification steps of MNP. SEM images are shown in Figure 1, while the results of DLS data and size histograms are given in Table 1 and Figure 2, respectively. The size of the MNP was increased after each modification step, as expected. The results are compatible with the images from SEM. In addition, the zeta potential results were correlated with the functionalisation of the MNPs. The results show that the surface modification of the MNPs was successfully performed.

### 3.3. Surface Characterisation

**Figure 3:**
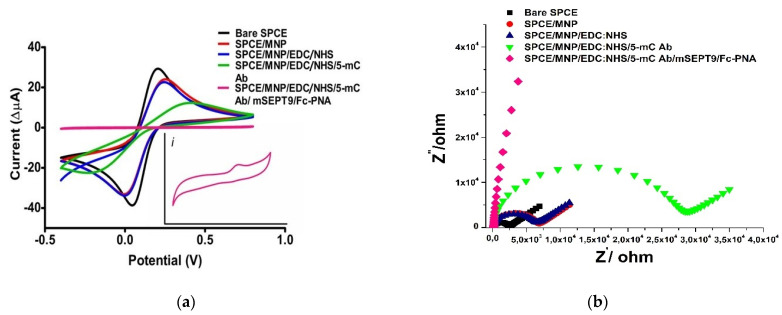
Surface characterisation results of the biosensor. (**a**) CV peaks between −0.5 and 1.0 mV s^−1^ scan rates (for SPCE/MNP/EDC/NHS/5-mC Ab/ mSEPT9/Fc-PNA was shown in the inset (i)]; (**b**) EIS [Electrochemical cell containing 0.1 M KCl and 5.0 mM HCF in 1X PBS buffer (pH 7.4) was used for measurements of Bare SPCE; SPCE/MNP; SPCE/MNP/EDC/NHS; SPCE/MNP/EDC/NHS/5-mC Ab steps. The electrochemical cell containing 0.1 M KCl in 1X PBS buffer (pH 7.4) was used for the measurement after addition of the mSEPT9/Fc-PNA hybridization].

CV and EIS measurements were performed for each modification step for surface characterisation of the biosensor. As shown in Figure 3a, the CV peaks decreased as a result of the surface (SPCE) modification with MNP and MNP/EDC/NHS. Subsequently, amino functional-MNPs were bound to the carboxyl group of 5-mC Ab by using EDC/NHS chemistry. mSEPT9 fragments were hybridised with Fc-PNA in a test tube, then the resulted hybrids were dropped to the electrode surface. According to the CV data, the cv peaks due to the bound Fc were dropped significantly. This can be explained by the fact that the massive groups blocked electron transfer on the electrode surface.

EIS measurements were also performed after each modification step. The EIS results were analysed using the Nyquist plot, which has lower and upper frequencies. The typical redox diffusion on the electrode surface appears as diagonal lines at lower frequencies, while the electron transfer resistance appears as semi-circular peaks at higher frequencies. In addition, the response of the EIS measurement was interpreted with an electrical circuit containing the solution resistance: Rs, the Warburg impedance: Zw, the double layer capacitance: Cdl, and the change in electron transfer resistance: Rct. As can be seen in Figure 3b, the resistance peaks grew after each modification step. This can be explained by the increasing bulky groups on the surface after each modification step. These groups prevented the interference of electrons on the electrode surface and the resistance values increased. This situation was expected and is consistent with the results from CV. The results show that the surface modification was successful. The numerical data for CV and the EIS measurements are shown in Table S3.

The detection principles of the proposed biosensor are shown in Scheme S1. The platform takes advantage of Fc-PNA probes for rapid and selective detection. Here, we developed a simple and novel biosensor for quantification of the mSEPT9 gene based on selective recognition features of Fc-PNA towards the target biomarker, which has the potential of early diagnosis of CRC. When hybridised with methylated DNA samples containing the mSEPT gene with Fc-PNA, the fc at the terminal end cannot reach the modified electrode surface with amino-modified MNPs with 5-mC-Ab. In this case, as the percentage of methylation increased, so did the DNA that hybridised to the PNA specifically designed for mSEPT9. This was observed as a decrease in electrical signals [38].

### 3.4. Optimization

Optimisation studies were performed for the design of the biosensor. First, the concentration of 5-mC Ab to be conjugated with MNP was searched. For this purpose, different amounts of 5-mC Ab (1.0; 5.0; 10 and 15 µg/mL) were added to MNP/EDC/NHS. The conjugate was applied to the electrode surface, and DPV measurements were performed after 50% mSEPT9/Fc-PNA solution was added to the surface. As shown in Figure S9a, the best current differences were obtained at 10 µg/mL of 5-mC Ab.

Additionally, the optimal concentration of mSEPT9 fragments for hybridization of Fc-PNA with different concentrations of 100% mSEPT9 was determined. The optimal mSEPT9 concentration was set at 5.0 nM using the current differential results (Figure S9b). In addition, the optimal time for immobilization of mSPET9/Fc-PNA was determined. The incubation of 1 h showed the best result for signal analysis (Figure S9c).

### 3.5. Analytical Properties of Biosensor

**Figure 4:**
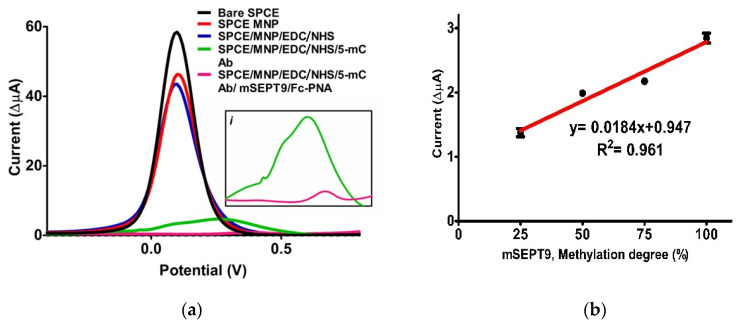
(**a**) DPV results of biosensor [Measurements for SPCE/MNP/EDC/NHS/5-mC Ab/and SPCE/MNP/EDC/NHS/5-mC Ab/Fc-PNA were shown in the inset (i)]. [Electrochemical cell containing 0.1 M KCl and 5.0 mM HCF in 1X PBS buffer (pH 7.4) was used for measurements of Bare SPCE; SPCE/MNP; SPCE/MNP/EDC/NHS; SPCE/MNP/EDC/NHS/5-mC Ab steps. The electrochemical cell containing 0.1 M KCl in 1X PBS buffer (pH 7.4) was used for the measurement after addition of the mSEPT9/Fc-PNA hybridization.] (**b**) Calibration graph of biosensor system.

DPV measurements were used to determine the calibration curve and other analytic parameters. The measurement results are shown in Figure 4a. The oxidation peaks were reduced similarly to the CV measurements.

The calibration curve was constructed using the DPV measurement. The current difference values between 5 and mC Ab with mSEPT9 fragment (25, 50, 75, and 100%)/Fc-PNA were subtracted from 0% mSEPT9 fragment/Fc-PNA (Figure 4b).

The detection limit (LOD) is the key analytical parameter for determining the lowest percentage of mSEPT9 that the biosensor can detect; 25% mSEPT9/Fc-PNA conjugated was added to the modified electrode surface and measurements (*n* = 10) were performed. The LOD value was calculated using the [(3x ± SD)/slope value] formula. All calculated analytical parameters are summarised in Table 2.

To further demonstrate the practical and clinical application of the developed biosensor, CRC patients and control plasma samples were collected. cfDNA was isolated from these samples. The biosensor detected SEPT9 methylation level in 20 ng/µL cfDNA extracted from plasma samples of CRC patients and control subjects, with surface stability exceeding that of other reported electrochemical platforms (Table 3). This may be related to the use of MNPs. In addition to the inherent magnetic force, superparamagnetic nanoparticles can also improve the analytical signal due to the oxidation/reduction processes, which can decrease the signal-to-noise ratio. Lin et al. fabricated a magnetic bead-based optical sensor with low detection limits (0.02 ng/µL) using HCT 116 cells [39]. Recently, Syedmoradi et al. reported an electrochemical system using SPCE electrodes that achieved a good linear range with a detection limit of 0.01 pM [40]. However, stability control was more difficult due to the lengthy surface modification steps. Regardless of achieving a slightly higher LOD concentration of mSEPT9, we believe the real strength of our approach is the simplicity and flexibility of the fabrication process. Most importantly, we modified the SPCE surface with MNPs modified with an anti-methyl cytosine antibody to achieve rapid accumulation of methylated DNA through specific immunoaffinity. In addition, our biosensor system worked in a shorter time than others. Therefore, its versatility, fast execution, and easy implementation at a low cost were remarkable.

### 3.6. Interferences

**Figure 5:**
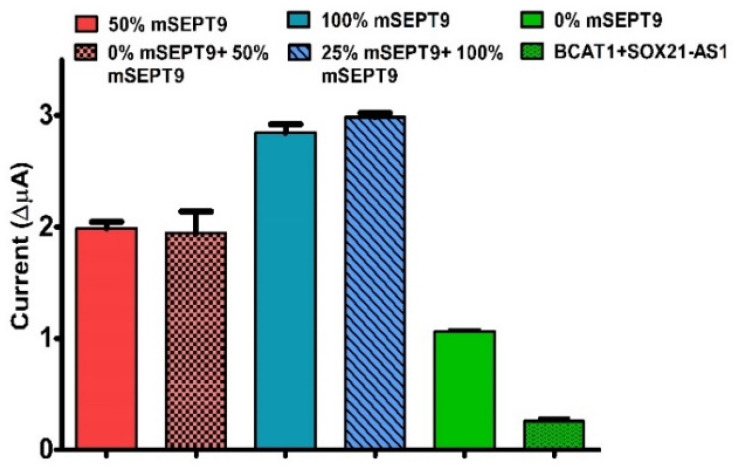
The response of the sensor towards different interferants including BCAT1 and SOX21-AS and various SEPT9 methylation levels.

The fragments of BCAT1 and SOX21-AS1 were selected as interference molecules because they are other methylated DNA structures found freely in the plasma of CRC patients [43]. A mixture of these fragments was applied over the biosensor to determine the specificity for mSEPT9. The results were compared with those obtained with 0% mSEPT9 fragments. The response of the sensor to the mixture was determined as 26.6%. Current literature indicates that the biosensor response to interfering molecules should be less than 30% [44]. Accordingly, the biosensor can be used specifically for the detection of mSEPT9 fragments. In addition, mixtures of mSEPT9 fragments (a mixture of 50% with 0% mSEPT9 and 25% with 100% mSEPT9) were prepared in different combinations and applied to the sensor surface to check the ability to detect various levels of methylation at the same time. The obtained results were compared with 50% and 100% mSEPT9 and the response of the biosensor was calculated as a percentage. The response of the sensor was determined as 97.67% for the mixture of 50% with unmethylated mSEPT9; whereas the mixture of 25% and 100% mSEPT9 produced a response level of 104.88% (Figure 5).

### 3.7. Clinical Sample Application and Confirmation

First, different mSEPT9 fragments (25% and 50%) were added to commercial human serum and hybridised with Fc-PNA. Then, these solutions were dropped onto the modified electrode surface. DPV measurements were carried out and the degree of methylation was calculated from the current values. In addition, the relative standard deviation (RSD) values were calculated and determined to be less than 5.0%. The results are summarised in Table 4.

**Figure 6:**
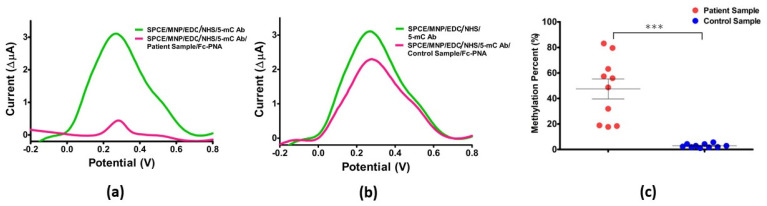
(**a**) Representative DPV peak forCRC plasma sample. (**b**) Representative DPV peak for control plasma sample. (**c**) mSEPT9 levels obtained from the developed sensor (***: *p* < 0.001).

In addition, plasma samples (cancer patient samples = 10; healthy samples = 10) were collected from CRC patients. Afterwards, cfDNAs were isolated and applied to the biosensor after hybridization with Fc-PNA. DPV peaks and mSEPT9 levels for CRC and control samples are given in Figure 6a–c.

Moreover, results were confirmed with RT-PCR and a methylated DNA quantification kit. All results are shown in Figure S10. In addition, the results obtained from different methods showed positive correlations with each other.

According to the results, mSEPT9 levels were detected in more than 50% of patients above stage II, depending on the histopathological stage of colon cancer. However, no difference was found between male and female patients. The developed sensor was shown to successfully discriminate mSEPT9 in control and patient samples. A statistically significant difference was found between the control and patient groups (*p* < 0.0001).

### 3.8. POC Device

**Figure 7:**
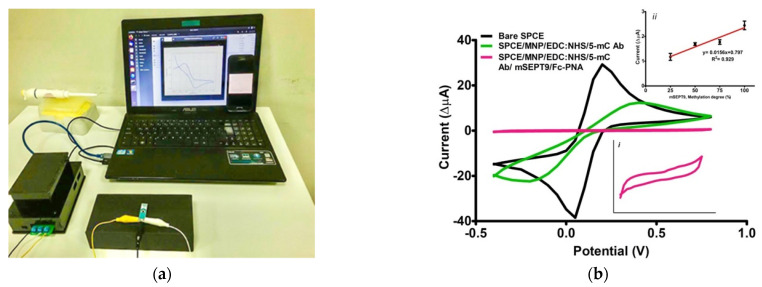
(**a**) Measurement display with design POC. (**b**) CV results with POC [Measurement for SPCE/MNP/EDC/NHS/5-mC Ab/mSEPT9/Fc-PNA was shown in the inset (i). Calibration curve was generated with POC was shown in the inset (ii)]. [Electrochemical cell containing 0.1 M KCl and 5.0 mM HCF in 1X PBS buffer (pH 7.4) was used for measurements of Bare SPCE; SPCE/MNP/EDC/NHS/5-mC Ab steps. The electrochemical cell containing 0.1 M KCl in 1X PBS buffer (pH 7.4) was used for the measurement after addition of the mSEPT9/Fc-PNA hybridization.].

The designed potentiostat and its connection to the computer are shown in Figure S11. The obtained data can also be transferred to the smartphone via Bluetooth connection (Figure 7a). CV measurements of the modification steps were performed using the POC device and the obtained peaks are shown in Figure 7b. As can be seen in Figure 6, the CV peaks decreased with each modification step. These results are as expected. In addition, a calibration graph was created using the CV peaks (Figure 7b (ii)). Compared to the results obtained with the PalmSens device, the CV peaks obtained with the developed device are similar. This shows that it is possible to use the POC device as a bedside diagnostic system.

## 4. Conclusions

In this work, an effective electrochemical DNA biosensor for the quantification of mSEPT9 was demonstrated by modifying SPCE with magnetic nanoparticles and using Fc-labelled PNA. Magnetic nanoparticles are preferred because they improve electrochemical responses and offer advantages such as biocompatibility and stability, ease of surface preparation, and high sensitivity. The application of PNA as electrochemical biosensors for DNA or RNA is promising and relatively new. In particular, PNAs labelled with a redox-active metal such as ferrocene offer serious advantages in terms of electrochemical systems. This highly selective MNP-based electrochemical sensor was developed for the rapid detection of the percentage of DNA methylation of the SEPT9 gene directly in DNA samples obtained from plasma samples. The readings of the developed system for mSEPT9 were linear between 25 and 100%, with a detection limit of 0.37%. The sensitivity of this sensor and the lowest detected methylation percentage are comparable to the most advanced DNA methylation sensors. Moreover, this biosensor was simple and did not require complicated processes such as PCR amplification. Therefore, this method has the potential to be developed into a powerful diagnostic tool for rapid and accurate methylation detection. The results show that this electrochemical system can be used in the early clinical diagnosis of CRC. However, the small number of patients and control samples in our study limits our conclusions regarding the suitability and adaptability of this system for clinical use.

## Figures and Tables

**Table 1:** Zeta potential and size measurement results for each modification steps of MNP.

	Zeta Potential (mV) ± SD	Size (d.nm) ± SD	PDI
MNP	10.30 ± 5.6	45.75 ± 18.8	0.15
MNP/TEOS	−42.40 ± 7.5	57.26 ± 20.9	0.11
MNP/TEOS/APTES	20.30 ± 5.8	64.78 ± 36.1	0.21
MNP/TEOS/APTES/EDC/NHS/5-mC Ab	24.20 ± 4.9	75.03 ± 43.5	0.21

**Table 2:** Analytical features of the sensing system.

Analytical Parameters	Values
Linear range (% methylation degree)	25–100
Slope	0.02
±SD * of slope	0.003
Intercept	0.95
±SD * of intercept	0.18
Correlation coefficient	0.96
Repeatability [±SD]	0.002
Coefficient of variation (%)	1.99
LOD (methylation degree, %)	0.37

* Standard deviation.

**Table 3:** An overview of existing mSEPT9 sensors in the literature.

Sensor Type	Sensor Design	Sample Type	LOD	Ref.
Optical	MB/5-mC Ab/mSEPT9/proteinase K/ LAMP primers/SYTO-9 dye	Clinical plasma	0.02 ng/μL	[39]
Electrical assay	CDs-LEG-FET/probe/mSEPT9/5-mC Ab	Tissue	2.0 ng	[41]
Optical	Prob DNA/mSEPT9/AgNC	Blood	7.6 × 10^−^^8^ M	[42]
Electrochemical	SPCE/GO/L-Arg/5-mC Ab/Fe_3_O_4_-TMC-Au- probe- mSEPT9	Plasma	0.01 pM	[40]
Proposed sensor (Electrochemical)	SPCE/NH_2_-MNP-5-mC Ab/Fc-PNA/mSEPT9	Plasma	0.37%	This work

MB: magnetic bead; LAMP: Loop-mediated isothermal amplification; CDs-LEG-FET: carbon dot-modified liquid exfoliated graphene field effect transistor; GO: Graphene oxide; L-Arg: poly(L-arginine); TMC: N-trimethylchitosan; AgNC: silver nanocluster.

**Table 4:** Application mSEPT9 in commercial human serum.

Added mSEPT9 (%)	Found mSEPT9 (%)	Recovery	RSD
50%	52.45 ± 0.03	104.89	1.56
75%	73.80 ± 0.02	98.41	0.93

## Data Availability

Not applicable.

## Supplementary Materials

The following supporting information can be downloaded at: https://www.mdpi.com/article/10.3390/bios12090736/s1, Figure S1: Potentiostat circuit; Figure S2: Electrical circuit printing; Table S1: The sequences used in this study. 5-methylcytosine (5-mC) is indicated with mC; Table S2: ITC measured thermodynamic parameters of the PNA-DNA interaction; Figure S3: Titration data for the hybridization between unmethylated SEPT9 fragment and PNA. Raw ITC data (top) and enthalpy change (down); Figure S4: Titration data for the hybridization between 100met_SEPT9 and PNA. Raw ITC data (top) and enthalpy change (down); Figure S5: Titration data for the hybridization between 75met_SEPT9 and PNA. Raw ITC data (top) and enthalpy change (down); Figure S6: Titration data for the hybridization between 50met_SEPT9 and PNA. Raw ITC data (top) and enthalpy change (down); Figure S7: Titration data for the hybridization between 25met_SEPT9 and PNA. Raw ITC data (top) and enthalpy change (down); Figure S8: VSM results; Table S3. Numerical data of CV and EIS measurements of biosensor; Scheme S1: Schematic depiction of the detection principle. Figure S9. Optimization steps of the proposed CRC diagnosis system. (a) 5-mC Ab concentration, (b) mSEPT9 concentration, and (c) mSEPT9/PNA incubation time; Figure S10: Comparative results for sensor, methylated DNA quantification kit (ELISA) and RT-PCR for (a) Patient samples, (b) Control samples; Figure S11: Images of the POC.
