# Supplementary material for: Magnetic Nanoparticle-Based Electrochemical Sensing Platform Using Ferrocene-Labelled Peptide Nucleic Acid for the Early Diagnosis of Colorectal Cancer
Source: Biosensors (Basel). 2022 Sep 7;12(9):736. doi: 10.3390/bios12090736 (PMC9496070; doi:10.3390/bios12090736)
Supplement: Supplementary file 1 [file biosensors-12-00736-s001.zip › biosensors-1881863-supplementary.pdf]

SUPPORTING INFORMATION

Magnetic Nanoparticle-based Electrochemical Sensing Platform using Ferrocene-labelled Peptide Nucleic Acid for the Early Diagnosis of Colorectal Cancer

Simge Balaban Hanoglu[1], Ezgi Man [1], Duygu Harmanci[2], Serife Tozan Ruzgar[2], Serdar Sanli[1], Nazim Arda Keles[3], Atakan Ayden[3], Bilge G. Tuna[4], Ozgul Duzgun[5], Omer Faruk Ozkan[5], Soner Dogan[3], Faezeh Ghorbanizamani[1], Hichem Moulahoum[1], Emine Guler Celik[6], Serap Evran[1,*] and Suna Timur[1,2,*]

[1]Department of Biochemistry, Faculty of Science, Ege University, 35100, Bornova, Izmir, Turkey.
[2]Central Research Test and Analysis Laboratory Application and Research Center, Ege University, 35100, Bornova, Izmir, Turkey.
[3]Department of Medical Biology, Yeditepe University, School of Medicine, Istanbul, Turkey.
[4]Department of Biophysics, Yeditepe University, School of Medicine, Istanbul, Turkey.
[5]Umraniye Training and Research Hospital, Surgical Oncology, Istanbul, Turkey.
[6]Department of Bioengineering, Faculty of Engineering, Ege University, 35100, Bornova, Izmir, Turkey.
*Correspondence: serap.evran@ege.edu.tr; suna.timur@ege.edu.tr

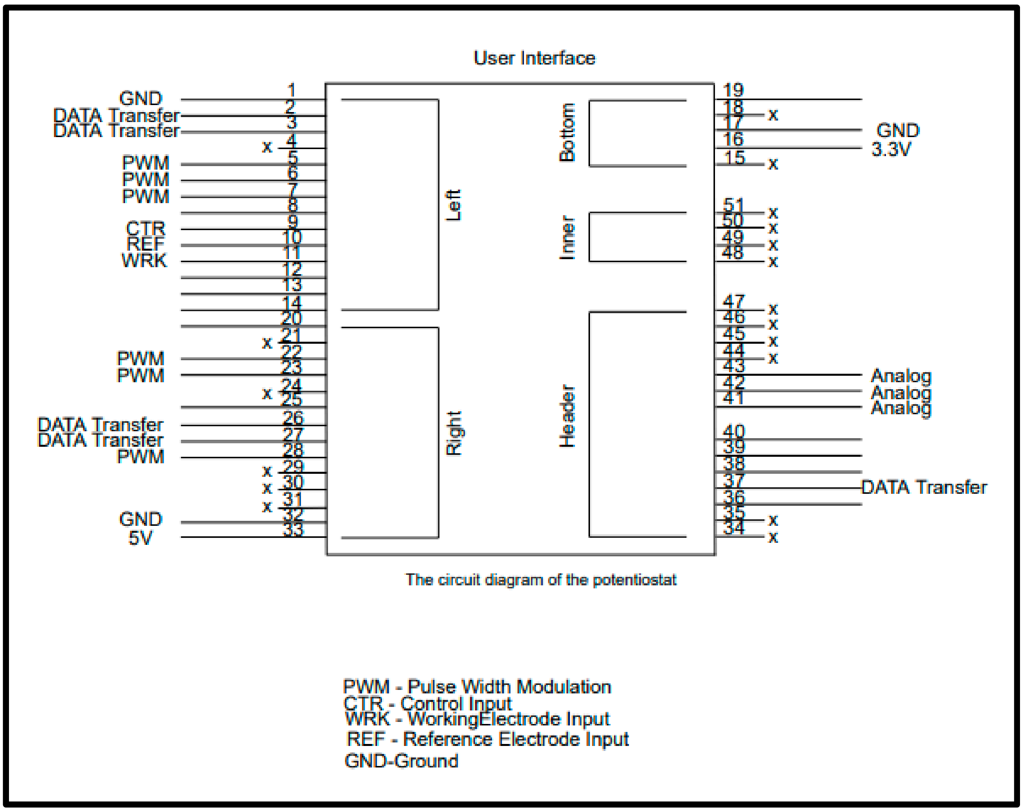

Figure S1. Potentiostat circuit.

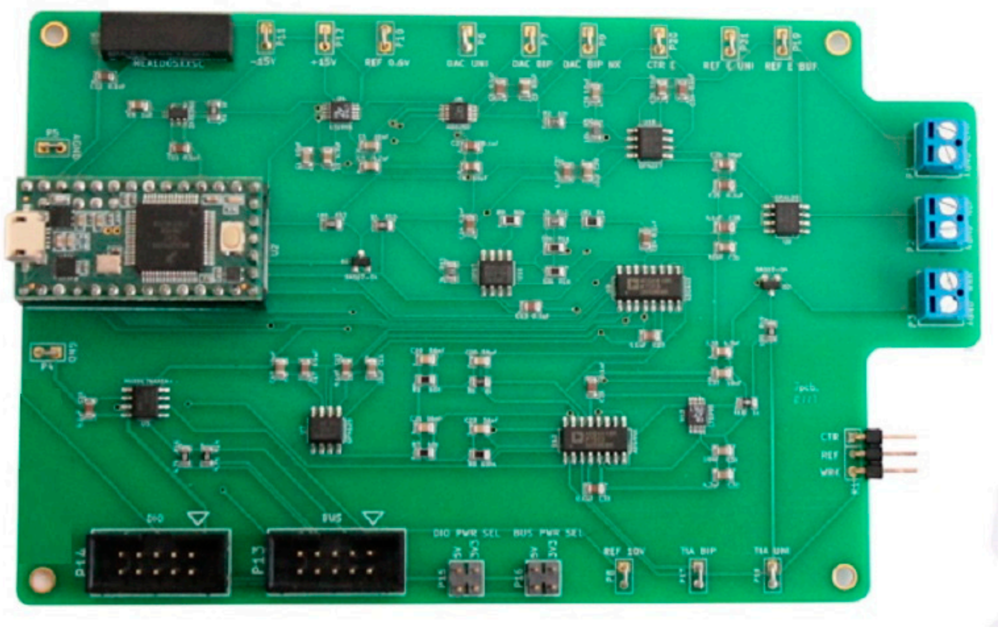

Figure S2. Electrical circuit printing.

Table S1. The sequences used in this study. 5-methylcytosine (5-mC) is indicated with mC.

**Name**	**Sequence 5′ → 3′**
SEPT9 amplicon 5	AGAAACGCACCATGGTTTGGATGCTGAGGGTGAGGGAAAGAGAGGA GTCAACAGTGGTGCCCGGAGACTTGGCTTGAGCAACTAGGTGGATGG TAGCACCGTTTCCTAAGATGAGGGGCTGTGGGAACTTGAGGGGCTGT GAAGGCTCTGCCACTGACGGACCCAGGGCTCGGCAGCTCTGGGAGGC CTACTTTGTGTCAGGCCACGCAGAGATAAGACCATCTGGGC
target SEPT9	TGGGAACTTGAGGGGCTGTGAAGGCTCTGCCACTGACGGACCCAGGG CTCGGCAGCTCTG
100met_SEPT9	TGGGAACTTGAGGGG**mC**TGTGAAGGCT**mC**TGCCA**mC**TGA**mC**GGACC **mC**AGGGCT**mC**GG**mC**AGCT**mC**TG
75met_SEPT9	TGGGAACTTGAGGGG**mC**TGTGAAGGCT**mC**TGCCA**mC**TGA**mC**GGACC CAGGGCT**mC**GGCAGCT**mC**TG
50met_SEPT9	TGGGAACTTGAGGGGCTGTGAAGGCTCTGCCA**mC**TGA**mC**GGACCCA GGGCT**mC**GGCAGCT**mC**G
25met_SEPT9	TGGGAACTTGAGGGGCTGTGAAGGCTCTGCCACTGACGGACCCAGGG CT**mC**GGCAGCT**mC**TG
BCAT1 promoter	TGCAATCCAGCC**mC**GCCA**mC**GTGTACTCGC**mC**GCCGCCT **mC**GGGCACTGCCCCAGGTCTTGCTG
SOX21-AS1 promoter	GCAGAG**mC**GCAGCAATGGATATAAATACAAATA**mC**AGCAGCAC**mC** AGC**mC**TGTCTTCTTGGCTC

Table S2. ITC measured thermodynamic parameters of the PNA-DNA interaction.

**DNA fragment**	**K_d (nM)**	**ΔH(kcal/mol)**	**ΔG(kcal/mol)**	**ΔS(kcal/mol)**
unmethylated SEPT9	26.5	-53.2	-10.3	-42.9
100met_SEPT9	85.5	-61.6	-9.65	-51.9
75met_SEPT9	20.8	-51.7	-10.5	-41.2
50met_SEPT9	30.4	-55.3	-10.3	-45.1
25met_SEPT9	9.49	-50.8	-10.9	-39.8

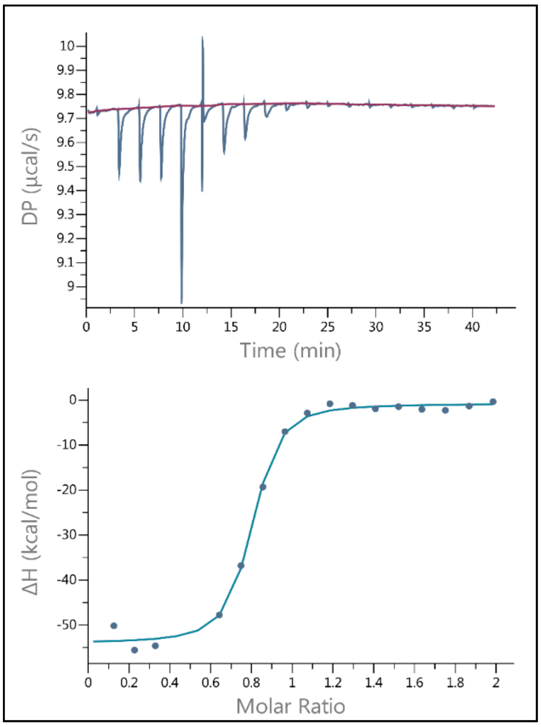

Figure S3. Titration data for the hybridization between unmethylated SEPT9 fragment and PNA. Raw ITC data (top) and enthalpy change (down).

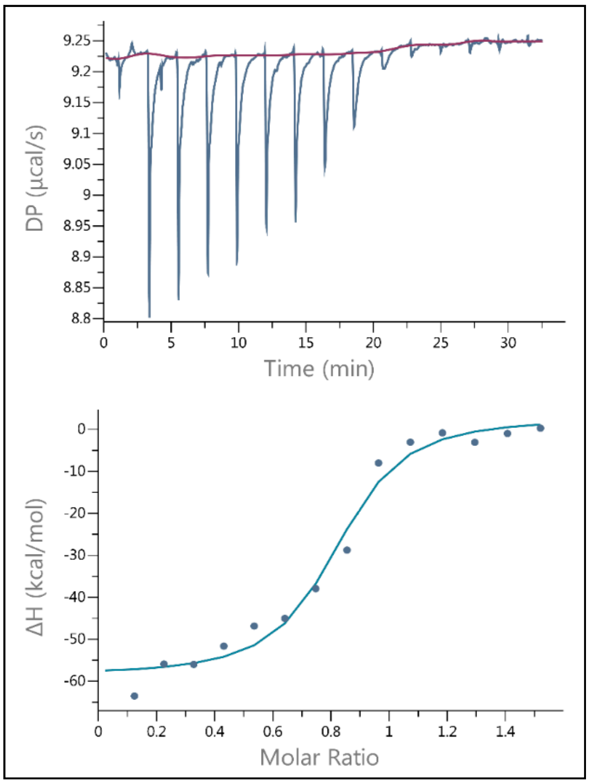

Figure S4. Titration data for the hybridization between 100met_SEPT9 and PNA. Raw ITC data (top) and enthalpy change (down).

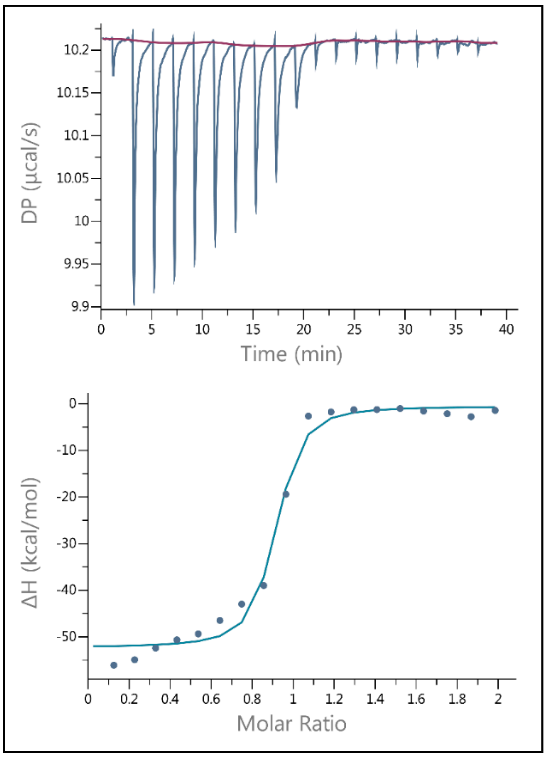

Figure S5. Titration data for the hybridization between 75met_SEPT9 and PNA. Raw ITC data (top) and enthalpy change (down).

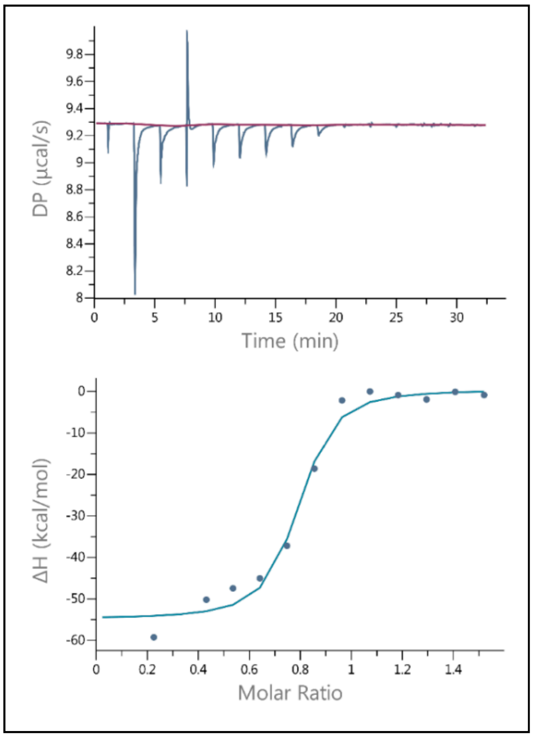

Figure S6. Titration data for the hybridization between 50met_SEPT9 and PNA. Raw ITC data (top) and enthalpy change (down).

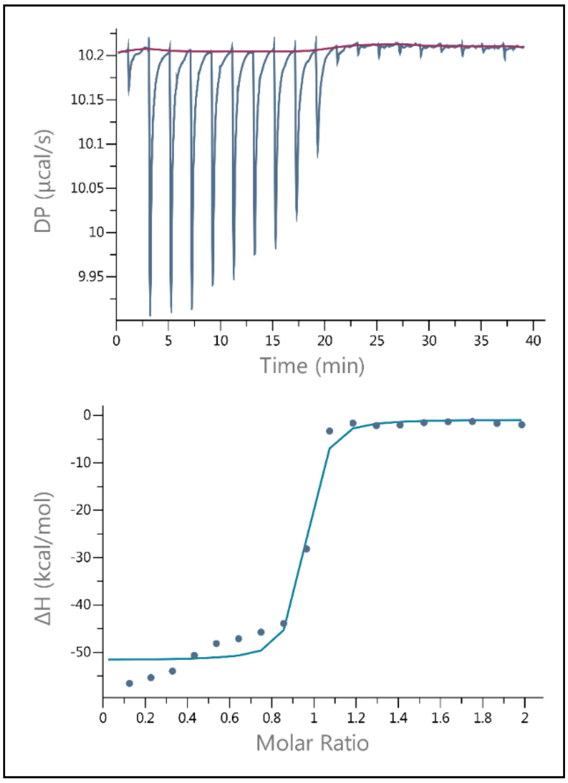

Figure S7. Titration data for the hybridization between 25met_SEPT9 and PNA. Raw ITC data (top) and enthalpy change (down).

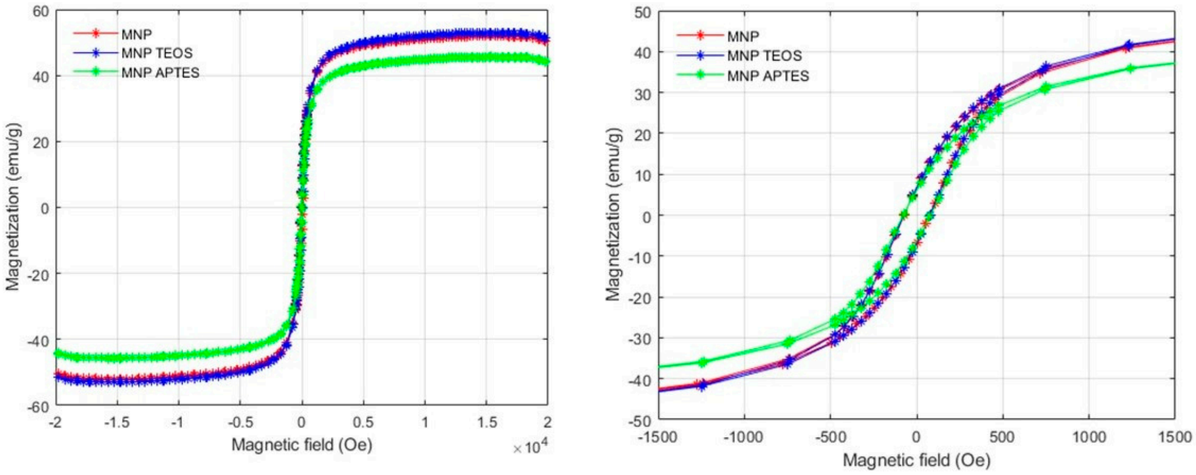

Figure S8. VSM results.

Table S3. Numerical data of CV and EIS measurements of biosensor.

	Cationic Current Values (μA)	Anionic Current Values (μA)	Ohm (Ω)
**Bare SPCE**	29.288	-38.676	2092
**SPCE/MNP**	24.009	-33.126	6192
**SPCE/MNP/EDC:NHS**	22.608	-32.811	5765
**SPCE/MNP/EDC:NHS/5-mC Ab**	12.318	-22.527	26560
**SPCE/MNP/EDC:NHS/5-mC Ab/mSEPT9/Fc-PNA**	0.310	-0.589	100000

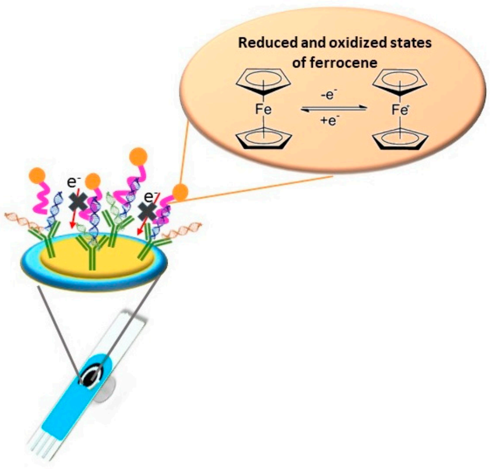

Scheme S1. Schematic depiction of the detection principle.

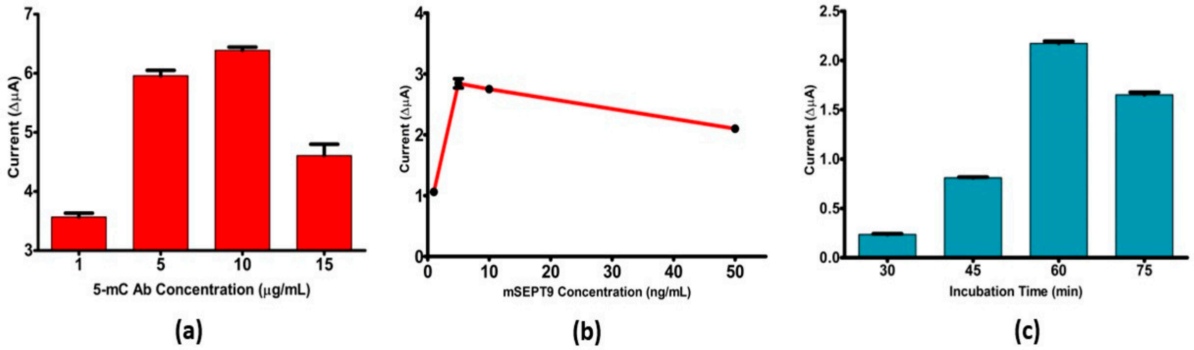

Figure S9. Optimization steps of the proposed CRC diagnosis system. (a) 5-mC Ab concentration, (b) mSEPT9 concentration, and (c) mSEPT9/PNA incubation time.

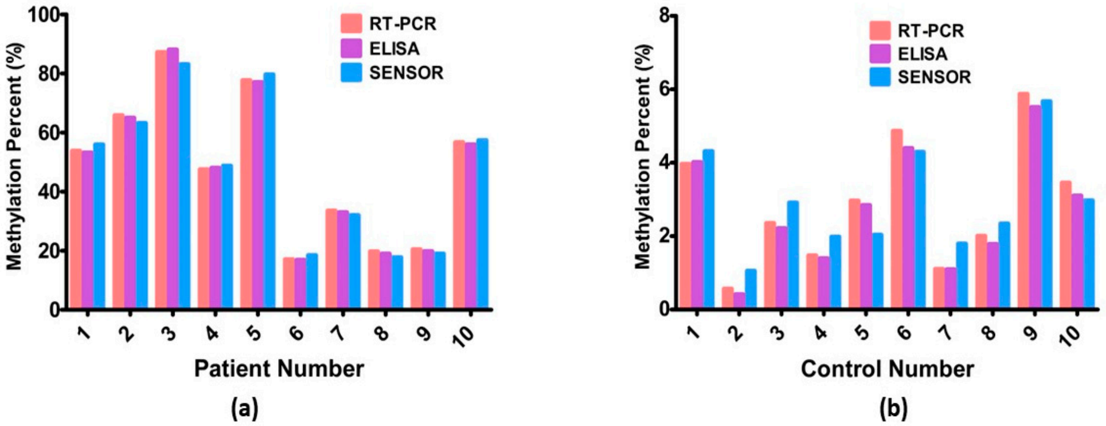

Figure S10. Comparative results for sensor, methylated DNA quantification kit (ELISA) and RT-PCR for (a) Patient samples (n=10), (b) Control samples (n=10).

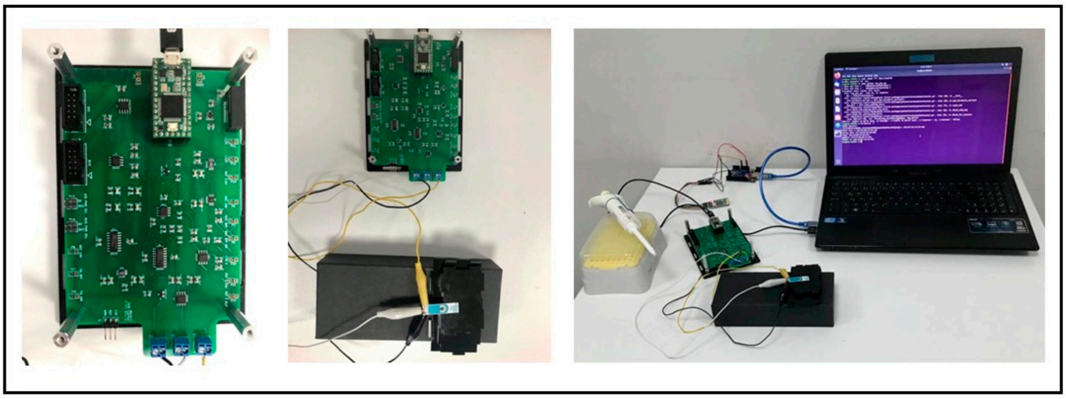

Figure S11. Images of the POC.
